# Network meta-analysis of 7 acupuncture therapies for knee osteoarthritis

**DOI:** 10.1097/MD.0000000000035670

**Published:** 2023-10-27

**Authors:** Weiwei Ma, Chao Yang Zhang, Xin Huang, Wei Cheng

**Affiliations:** a School of Acupuncture-Moxibustion and Orthopedics, Hubei University of Chinese Medicine, Wuhan, China; b Wuhan Hospital of Traditional Chinese Medicine, Wuhan, China.

**Keywords:** acupuncture, knee osteoarthritis, network meta-analysis, randomized controlled study, systematic review

## Abstract

**Objective:**

With the progression of society aging demographic, the prevalence of knee osteoarthritis (KOA) continues to rise steadily, exerting a significant impact on individuals’ quality of life. Acupuncture therapy has garnered extensive utilization in the management of osteoarthritis; however, a comprehensive systematic review integrating acupuncture with traditional Chinese medicine remains absent. This study compared the clinical efficacy of 7 acupuncture methods (electroacupuncture, conventional acupuncture, warm needle, floating needle, fire needle, needle knife, and silver needle) for the treatment of KOA through a network meta-analysis.

**Methods:**

This study examined the databases—PubMed, EMbase, The Cochrane Library, the China Biology Medicine, Chinese Journal Full-text Database, Wanfang Database, and VIP Database—for randomized controlled trials of the 7 methods for KOA treatment. The search time spanned from the database establishment to March 5, 2022. The primary outcome indicator was the total effective rate, and the secondary outcome indicator was the visual analog scale. After the layer-by-layer screening, the quality of the literature was assessed using the Cochrane systematic reviewer manual 5.1.0 bias risk assessment tool for randomized controlled trials. After data extraction, the R4.0.1 software was used for network meta-analysis.

**Results:**

Based on the network meta-analysis, the ranking of interventions based on the surface under the cumulative ranking curve for the total effective rate is as follows: silver needle (0.99) > floating needle (0.97) > needle knife (0.66) > fire needle (0.56) > warm needle (0.44) > conventional acupuncture (0.35) > electroacupuncture (0.13). Regarding the improvement in visual analog scale scores, the surface under the cumulative ranking curve ranking is as follows: silver needle (0.97) > conventional acupuncture (0.67) > needle knife (0.64) > floating needle (0.51) > warm needle (0.44) > fire needle (0.14) > electroacupuncture (0.09).

**Conclusion:**

Based on the network meta-analysis, silver needle therapy emerged as the most efficacious and analgesic intervention for KOA. Nevertheless, given the notable variations in the quality and quantity of studies encompassing diverse treatment modalities, the findings of this research necessitate further substantiation through forthcoming high-quality multicenter, large-sample, randomized double-blind trials.

## 1. Introduction

Osteoarthritis (OA)—a degenerative disease of synovial joints—is characterized by cartilage destruction, extracellular matrix degradation, and synovitis.^[1]^ OA not only leads to joint pain, deformity, and dysfunction^[2]^ but also significantly increases the risk of cardiovascular events,^[3]^ lower extremity deep venous thromboembolism,^[4]^ and all-cause mortality.^[5]^ The current global incidence of OA exceeds 300 million.^[6]^ With the increasing global aging, this number will continue to rise in the future, consuming a substantial amount of medical and social security resources. Modern medical treatment for OA continues to focus on reducing disease risk, slowing disease progression, and eliminating pain.^[7]^ Clinically used non-steroidal anti-inflammatory drugs cannot fundamentally reverse cartilage degeneration, and their side effects are concerning.^[8]^ Although intra-articular injection of glucocorticoids can temporarily relieve pain,^[9]^ its long-term efficacy is unclear,^[10]^ and long-term application of hormones accelerate articular cartilage degeneration.^[11]^ Surgical treatment is only suitable for late-stage patients with severe loss of joint function, and the pain caused by surgical risks and postoperative complications cannot be ignored. Therefore, a minimally invasive, low-risk treatment that can prevent articular cartilage degeneration in OA is urgently required.

Acupuncture therapy, a traditional Chinese treatment method, has had a significant effect on the treatment of knee osteoarthritis (KOA). The clinical treatment effect of acupuncture therapy is better than that of conventional therapy such as oral administration.^[12]^ Many scholars have conducted evidence-based medical research. Tian et al^[13]^ published a systematic review in 2022 and believed that acupuncture has a beneficial effect in relieving pain and improving functional activities. This therapy can be used as a useful alternative therapy for KOA patients. According to controlled clinical trials conducted by Ye et al,^[14]^ acupuncture had a short-term effect on KOA than the control group,acupuncture can effectively alleviate the clinical symptoms of KOA. However, the current landscape of clinical management for KOA encompasses various types of acupuncture treatments, with a dearth of direct comparative evidence regarding their respective effectiveness. This scarcity poses challenges in determining the optimal treatment approach in clinical practice. Network meta-analysis, as an advanced statistical methodology, facilitates the comparison and ranking of intervention effectiveness for a given disease. Consequently, the present study employs network meta-analysis to compare the therapeutic effects of diverse acupuncture treatments for KOA, with the objective of furnishing evidence-based guidance for optimizing treatment selection in clinical practice.

## 2. Information and methods

### 2.1. Protocol and registration

The meta-analysis protocol was registered with PROSPERO (CRD42022361063). This meta-analysis was reported according to the Preferred Reporting Items for Systematic Review and Meta-Analysis statement and recommendations.

### 2.2. Literature search

Computer Search English databases: PubMed, EMbase, The Cochrane Library, and China biology medicine; Chinese databases: Chinese journal full-text database, Wanfang, and VIP. Search terms include acupuncture, floating needle, needle, electroacupuncture, warm acupuncture, fire needle, silver needle, needle knife, osteoarthritis, KOA, randomized, and controlled. The retrieval strategy was constructed by combining subject words with free words, and the retrieval time limit was from the database establishment to March 5, 2022.

### 2.3. Inclusion and exclusion criteria for articles

#### 2.3.1. *Inclusion criteria*.

Research type: randomized controlled trial (RCT); literature language was limited to Chinese and English; Study subjects: anyone who met the American College of Rheumatology (1986, 1995, 2001) diagnostic criteria for OA of the knee, the Diagnostic Process and Treatment Strategy for Rheumatic Diseases or The accepted diagnostic criteria for OA of the knee, such as the guidelines for the diagnosis and treatment of OA issued by the Orthopedic Branch of the Chinese Medical Association (2007, 2010), were included in the study; Intervention measures: the treatment group for ordinary acupuncture, electroacupuncture, warm needle, float needle, fire needle, and silver needle, needle knife; the control group served as the comparison of the 7 acupuncture methods described in the treatment group; 2 outcome indicators: total effective rate, visual analog scale (VAS).

#### 2.3.2. Exclusion criteria.

Treatment group and control group in addition to the aforementioned 7 kinds of acupuncture and conventional treatment measures or Western medicine and other treatment interventions; non-Chinese, English literature, and only abstracts without full-text literature; unable to obtain full-text and repeated publications; data is wrong or incomplete; non-RCT studies: case reports and cross-sectional studies, longitudinal studies such as cohort studies, case-control studies, and systematic reviews, meta-analysis, reviews, experience summary, theoretical discussion, animal experiments, and cell tissue studies.

### 2.4. Literature screening and data extraction

The literature extracted from the database was imported into Note Express 3.0 software for literature management. First, duplicate literature was eliminated by examining duplicate literature, followed by eliminating literature that did not meet the inclusion criteria, as determined by reading the title and abstract. Finally, the full text of the literature that met the requirements was downloaded and further read to screen out qualified literature. A unified data extraction table was designed. To ensure data accuracy and study validity, 2 researchers extracted the relevant data. The extracted contents primarily included the author of the included study, the patient age, the treatment time, the intervention measures, and the outcome indicators. After completing the data extraction, the data were integrated and cross-checked. In the outlined process, if there were differences, third-party experts with many years of experience in evidence-based medicine jointly judged to determine the final result.

### 2.5. Quality evaluation of the included studies

Two researchers evaluated the quality of the included studies according to the Cochrane bias risk assessment tool recommended by the Cochrane systematic evaluation manual version 5.1.^[15]^ Cochrane risk bias assessment included 7 aspects: random sequence generation, allocation concealment, blinding of participants and researchers, blinding of evaluators, completeness of outcomes, selective reporting of results, and other sources of bias. Each item was evaluated for its low risk, high risk, and uncertainty. The quality evaluation of the literature was conducted independently by 2 researchers, and discrepancies were resolved through discussion.

### 2.6. Statistical analysis

The network meta-analysis employed the Bayesian random effect model. The “gemtc” package in R4.0.1 software was utilized to invoke the Markov Chain Monte Carlo method for determining the probability ranking of each intervention. The odds ratio (OR) served as the effect measure for count data, while the weighted mean difference was employed for continuous data. The 95% confidence interval (CI) was utilized for interval estimation. A comparison-adjusted funnel plot was deployed to identify small sample effects and conduct publication bias assessment. To rank the effectiveness of different interventions for each outcome measure, the surface under the cumulative ranking curve (SUCRA) was employed.

## 3. Results

### 3.1. Literature search results

**Figure 1.**
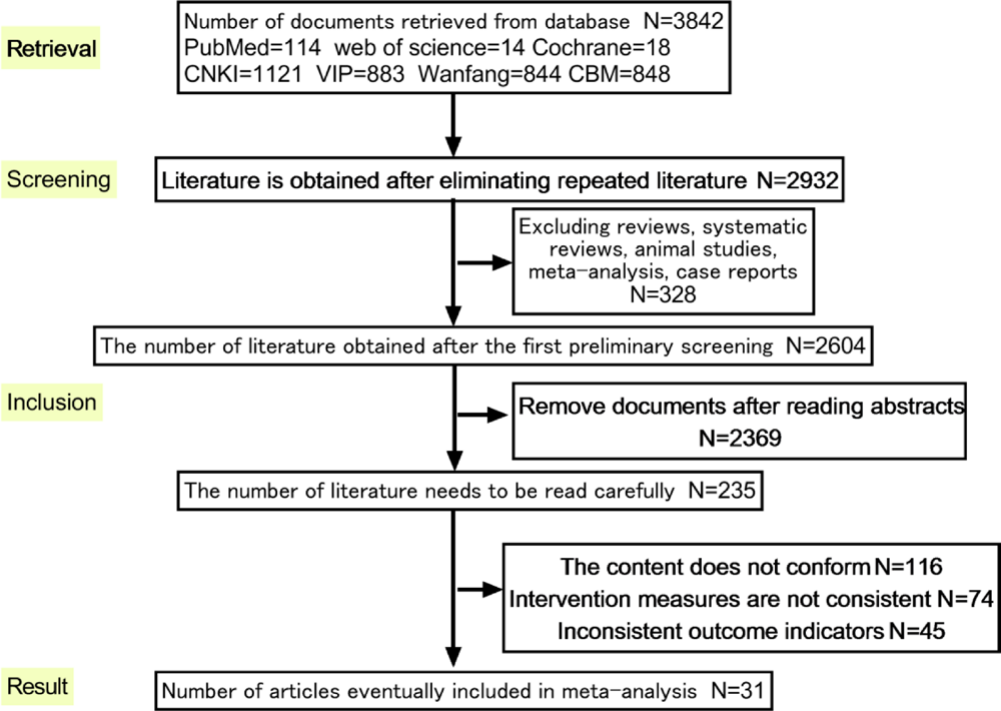
The literature selection process.

A comprehensive search yielded a total of 3842 articles from prominent authoritative databases both domestically and internationally, comprising 3696 Chinese articles and 146 English articles (PubMed = 114, Web of Science = 14, Cochrane Library = 18, CNKI = 1121, China biology medicine = 848, Wanfang Data = 844, VIP = 833). Following rigorous assessment, 31 articles were deemed eligible for inclusion.^[16–45]^ The literature screening process and outcomes are visually presented in Figure 1.

### 3.2. Basic characteristics of the included studies

A total of 2488 KOA patients were included in the 31 RCT, involving conventional acupuncture versus float needle (three items), fire needle versus Conventional acupuncture (five item), conventional acupuncture versus electroacupuncture (two items), warm needle versus electroacupuncture (two items), warm needle versus fire needle (three items), warm needle versus conventional acupuncture (two items), warm needle versus silver needle (four items), conventional acupuncture versus silver needle (one item), acupotomy versus conventional acupuncture (three items), acupotomy versus electroacupuncture (one item), acupotomy versus silver needle (one item), float needle versus electroacupuncture (one item), electroacupuncture versus fire needle (one item), fire needle versus electroacupuncture (two items). In terms of patient dropout, 7 studies have reported dropout, but the outcomes were not analyzed intentionally. Table 1 presents the basic characteristics of the included studies.

**Table 1:** Basic characteristics of the included literature.

Authory	Interventions		Sample size		Average age/yr		Outcome index	Treatment/wk
	T	C	T	C	T	C		
Tian Qi Wang	A	B	30	30	58.89 ± 6.75	59.70 ± 7.36	①②	4
Liu Na	B	A	41	37	60.98 ± 7.56	61.72 ± 8.05	①②	4
Gao Jie	A	C	34	35	37.35 ± 10.83	38.86 ± 12.12	①②	8
Chen Yinghua	D	B	59	58	59.20 ± 5.48	59.73 ± 5.56	①②	2
Huang Zhi Gang	D	A	50	50	52.21 ± 8.65	52.21 ± 8.65	①②	3
Zhang Yan	D	B	35	35	59.23 ± 1.28	59.52 ± 1.06	①②	2
Xiao Binbin	D	B	35	35	56.9 ± 8.62		①②	2
Zhang Guoyan	E	B	32	32	57.22 ± 2.74	58.02 ± 2.33	①②	2
Zhang Jing	E	B	34	34	54.30 ± 7.17	53.25 ± 6.75	①②	3
Zheng Mei	E	B	35	35	62.39 ± 8.004	61.41 ± 8.203	①②	2
Cheng Gang	E	B	38	40	52.70 ± 5.23	53.50 ± 6.41	①	4
Zhou Liyan	A	E	60	60	65 ± 6	63 ± 6	①②	4
Lu Dejian	E	B	48	49	54.11 ± 9.46	60.81 ± 10.09	②	
Ho Tin Fung	E	A	55	57	58 ± 5	56 ± 5	①②	4
Zhang Pingping	E	C	50	50	50.26 ± 2.16	50.24 ± 2.14	①②	4
Fan Chunlan	E	C	54	54	58 ± 6.2	56 ± 8.4	①	4
Chen Kaiming	G	F	35	35	69.21 ± 0.87	71.12 ± 0.74	①	3
Deng Chang Heng	C	A	59	59	61.95 ± 1.50	61.83 ± 1.09	①	4
Li Hong’er	C	B	46	46	69.9 ± 5.7	69.5 ± 5.4	①	4
Wang Ming ming	C	B	32	32	60.5 ± 5.4	61.1 ± 5.3	①②	6
Liu Jun	G	C	60	60	51.50 ± 5.04	51.32 ± 4.95	①②	3
Qin Wei qiang	G	C	48	48	60.2 ± 2.6	57.2 ± 4.6	①②	4
Zeng Qing zhou	G	C	23	23	54.5 ± 8.2	56.5 ± 8.5	①②	5
Li Zhen	G	C	40	40	55.34 ± 5.68	54.87 ± 4.82	①②	4
Duan Wei min	G	C	45	45	60.17 ± 5.83	60.81 ± 6.54	①②	2
Liu Jian liang	G	B	40	40	62 ± 5	65 ± 5	①②	3
Hung Hon Ching	F	B	31	30	56.20 ± 8.32	59.37 ± 9.63	①②	3
Lin Fu Chang	F	B	30	30	54.68 ± 6.39	55.83 ± 6.83	①②	3
Xiu Zhong biao	F	B	40	40	52.6	53.2	①②	2
Quan Ke	F	A	25	25	56.4 ± 6.1		①②	2

A: electroacupuncture; B: acupuncture; C: warm needling; D: floating needle; E: fire needle; F: needle knife; G: silver needle; ①: efficiency rate; ②: VAS score; T: treatment group;C: control group.

### 3.3. Evaluation of the quality of the literature

**Figure 2.**
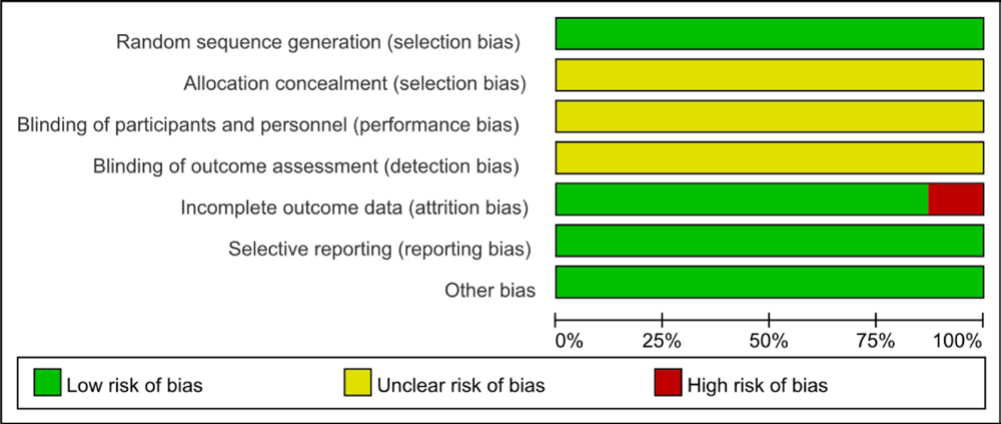
Publication bias graph.

A total of 31 studies were included. In terms of the random allocation method, 8 low-risk studies were assigned randomly through a random number table or computer randomization. The remaining 23 studies referred only to randomness without specifying a random allocation scheme. Thirty-one studies did not mention hiding and blinding random allocation schemes; the research data were complete. Because of the inability to obtain the registration plan of 31 studies, selective reporting of research results was judged based on the method of viewing the literature methodology section and the results section, and 31 studies were fully reported as low risk. All studies described baseline comparability and no other obvious biases were observed in these low-risk studies; other sources of bias were unclear. Figure 2 depicts the bias risk assessment of the included studies.

### 3.4. Treatment effectiveness

#### 3.4.1. Evidence network.

**Figure 3.**
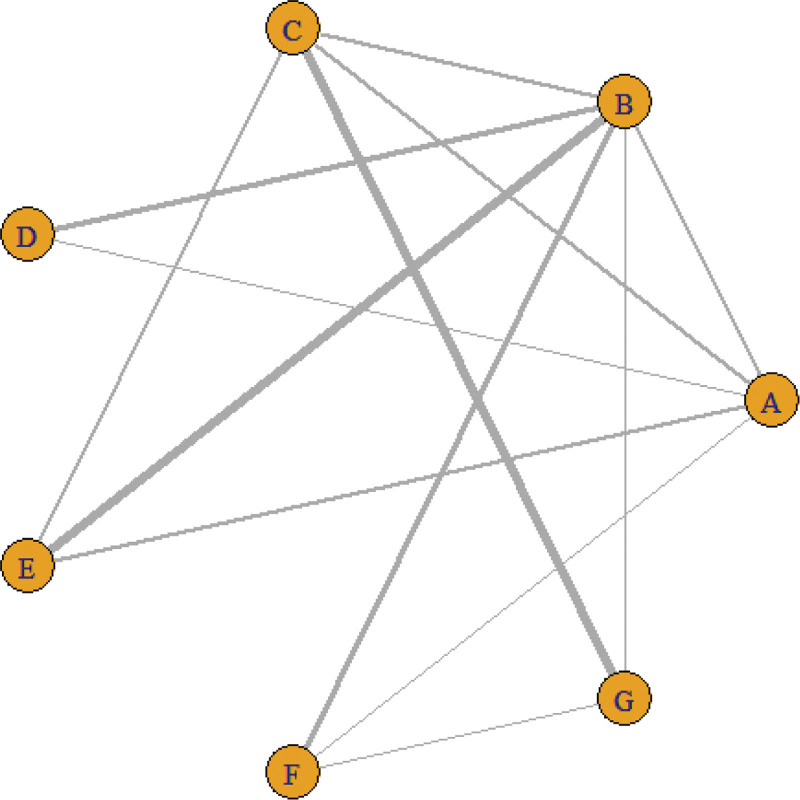
Efficient network relationship diagram. Notes: The thicker and darker the line, the greater the correlation between the 2 studies.

A total of 30 studies reported the effective rate of treatment, involving 7 kinds of acupuncture, 13 direct comparisons, and forming 7 closed loops. Figure 3 shows network relationships between the interventions. The results demonstrated that the number of studies on the comparison between conventional acupuncture and fire acupuncture was the most (5 RCTs), and the number of studies on the comparison between warm acupuncture and silver acupuncture was the most (432 cases), See Table 2 for details.

**Table 2:** Treatment effectiveness network evidence table.

Interventions		Sample size		Average age/yr		Treatment/wk
T	C	T	C	T	C	
A	B	71	67	59.19 ± 5.75	59.20 ± 7.66	4
A	C	93	94	37.35 ± 10.83	38.86 ± 12.12	8
D	B	129	128	58.80 ± 4.48	59.13 ± 4.56	2
D	A	50	50	53.21 ± 7.65	52.21 ± 6.65	3
E	B	139	141	56.12 ± 2.64	57.12 ± 1.33	2
A	E	115	117	64.8 ± 6	64 ± 6	4
E	C	94	94	51.16 ± 2.06	51.14 ± 2.04	4
G	F	35	35	70.21 ± 0.87	70.12 ± 0.74	3
C	B	78	78	69.9 ± 5.7	69.5 ± 5.4	4
G	C	216	216	52.10 ± 5.04	52.32 ± 5.15	3
G	B	40	40	62 ± 5	65 ± 5	3
F	B	101	100	57.20 ± 8.32	58.87 ± 9.63	3
F	A	25	25	56.4 ± 6.1		2

A: electroacupuncture; B: acupuncture; C: warm needling; D: floating needle; E: fire needle; F: needle knife; G: silver needle.

#### 3.4.2. Direct comparison of meta-analysis results and consistency tests.

**Figure 4.**
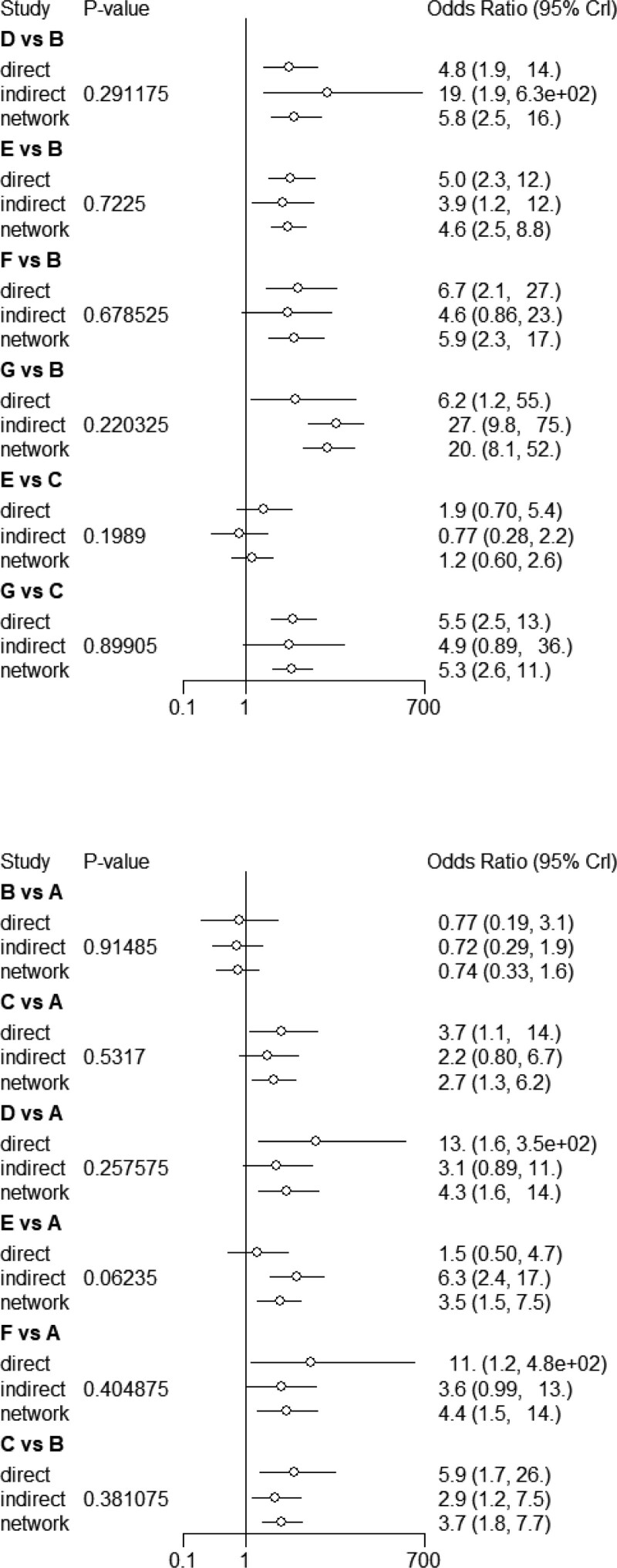
Efficient direct comparison and inconsistency test.

A meta-analysis was performed on the literature that was compared in pairs with efficiency as the main index. The results demonstrated that float needle was better than electroacupuncture (OR = 4.8, 95%CI [1.9,14]), fire needle was better than conventional acupuncture (OR = 5.0, 95%CI [2.3,12]), needle knife was better than conventional acupuncture (OR = 6.1, 95%CI [2.1,24]), silver needle was better than conventional acupuncture (OR = 6.2, 95%CI [1.2,55]), fire needle was better than warm needle (OR = 1.9, 95%CI [0.70,5.4]). Silver needle was superior to warm needle (OR = 5.5, 95%CI [2.5,13]), electroacupuncture was superior to conventional acupuncture (OR = 0.77, 95%CI [0.19,3.1]), warm needle was superior to electroacupuncture (OR = 3.7, 95%CI [1.1,14]), floating needle was superior to electroacupuncture (OR = 13, 95%CI [1.6,3.5 + 02]), fire needle was superior to electroacupuncture (OR = 1.5, 95%CI [0.5,4.7]). Acupotomy was superior to electroacupuncture (OR = 11, 95%CI [1.2,4.8e + 02]) and warm needle was superior to conventional acupuncture (OR = 5.9, 95%CI [1.7,26]). The comparison results were tested at *P* > .05, indicating that the direct comparison and indirect comparison of each acupuncture therapy had a good consistency and that the data analysis was conducted using the consistency model. The results are shown in Figure 4 and Table 3.

**Table 3:** Treatment effectiveness direct comparison between different acupuncture methods.

Treatment group	Control group	OR	95% Confidence interval
Float needle	Electroacupuncture	4.8	95%CI (1.9,14)
Fire needle	Conventional acupuncture	5.0	95%CI (2.3,12)
Needle knife	Conventional acupuncture	6.2	95%CI (1.2,55)
Fire needle	Warm needle	1.9	95%CI (0.70,5.4)
Silver needle	Warm needle	5.5	95%CI (2.5,13)
Electroacupuncture	Conventional acupuncture	0.77	95%CI (0.19,3.1)
Warm needle	Electroacupuncture	3.7	95%CI (1.1,14)
Floating needle	Electroacupuncture	13	95%CI (1.6,3.5 + 02)
Fire needle	Electroacupuncture	1.5	95%CI (0.5,4.7)
Acupotomy	Acupotomy	11	95%CI (1.2,4.8e + 02)
Warm needle	Conventional acupuncture	5.9	95%CI (1.7,26)

#### 3.4.3. Reticulation meta-analysis.

Effectiveness A network meta-analysis using a consistency model yielded the following results: Electroacupuncture was better than routine acupuncture (OR = 1.34, 95%CI [0.63,2.78]); warm needle was better than electroacupuncture (OR = 0.36, 95%CI [0.16,0.78]); float needle was better than electroacupuncture (OR = 0.23, 95%CI [0.08,0.63]); fire needle was better than electroacupuncture (OR = 0.28, 95%CI [0.13,0.62]); and acupotomy was better than electroacupuncture (OR = 0.23, 95%CI [0.07,0.65]). Silver needle was superior to electroacupuncture (OR = 0.07, 95%CI [0.02,0.19]); warm needle was superior to conventional acupuncture (OR = 0.27, 95%CI [0.13,0.57]); floating needle was superior to conventional acupuncture (OR = 0.17, 95%CI [0.07,0.4]); fire needle was superior to conventional acupuncture (OR = 0.21,95%CI [0.11,0.4]); and acupotomy was superior to conventional acupuncture (OR = 0.17,95%CI [0.06,0.43]). Silver needle was better than conventional acupuncture (OR = 0.05,95%CI [0.02,0.12]), silver needle was better than warm needle (OR = 0.19,95%CI [0.09,0.38]), silver needle was better than fire needle (OR = 0.23,95%CI [0.09,0.61]), silver needle was better than needle knife (OR = 0.3,95%CI [0.09, 0.96]). The remaining comparisons were not statistically significant (see Table 4 for details).

**Table 4:** Results of efficient mesh meta-analysis.

A	0.74 (0.36, 1.58)	2.79 (1.28, 6.07)	4.43 (1.58, 12.98)	3.51 (1.61, 7.78)	4.31 (1.53, 13.6)	14.81 (5.37, 42.13)
1.34 (0.63, 2.78)	B	3.77 (1.87, 7.51)	5.89 (2.53, 15.23)	4.67 (2.48, 8.99)	5.89 (2.33, 15.97)	19.85 (8.1, 51.56)
0.36 (0.16, 0.78)	0.27 (0.13, 0.53)	C	1.57 (0.55, 4.92)	1.24 (0.61, 2.56)	1.56 (0.55, 4.75)	5.27 (2.61, 11.28)
0.23 (0.08, 0.63)	0.17 (0.07, 0.4)	0.64 (0.2, 1.82)	D	0.79 (0.27, 2.32)	0.99 (0.27, 3.63)	3.38 (0.94, 12.4)
0.28 (0.13, 0.62)	0.21 (0.11, 0.4)	0.81 (0.39, 1.63)	1.27 (0.43, 3.76)	E	1.25 (0.44, 3.86)	4.26 (1.65, 11.26)
0.23 (0.07, 0.65)	0.17 (0.06, 0.43)	0.64 (0.21, 1.83)	1.01 (0.28, 3.69)	0.8 (0.26, 2.25)	F	3.33 (1.05, 10.89)
0.07 (0.02, 0.19)	0.05 (0.02, 0.12)	0.19 (0.09, 0.38)	0.3 (0.08, 1.06)	0.23 (0.09, 0.61)	0.3 (0.09, 0.96)	G

A: electroacupuncture; B: acupuncture; C: warm needling; D: floating needle; E: fire needle; F: needle knife; and G: silver needle.

#### 3.4.3. Reticulation meta-analysis.

Effectiveness A network meta-analysis using a consistency model yielded the following results: Electroacupuncture was better than routine acupuncture (OR = 1.34, 95%CI [0.63,2.78]); warm needle was better than electroacupuncture (OR = 0.36, 95%CI [0.16,0.78]); float needle was better than electroacupuncture (OR = 0.23, 95%CI [0.08,0.63]); fire needle was better than electroacupuncture (OR = 0.28, 95%CI [0.13,0.62]); and acupotomy was better than electroacupuncture (OR = 0.23, 95%CI [0.07,0.65]). Silver needle was superior to electroacupuncture (OR = 0.07, 95%CI [0.02,0.19]); warm needle was superior to conventional acupuncture (OR = 0.27, 95%CI [0.13,0.57]); floating needle was superior to conventional acupuncture (OR = 0.17, 95%CI [0.07,0.4]); fire needle was superior to conventional acupuncture (OR = 0.21,95%CI [0.11,0.4]); and acupotomy was superior to conventional acupuncture (OR = 0.17,95%CI [0.06,0.43]). Silver needle was better than conventional acupuncture (OR = 0.05,95%CI [0.02,0.12]), silver needle was better than warm needle (OR = 0.19,95%CI [0.09,0.38]), silver needle was better than fire needle (OR = 0.23,95%CI [0.09,0.61]), silver needle was better than needle knife (OR = 0.3,95%CI [0.09, 0.96]). The remaining comparisons were not statistically significant (see Table 4 for details).

#### 3.4.4. Comparison of the efficacy of different interventions.

**Figure 5.**
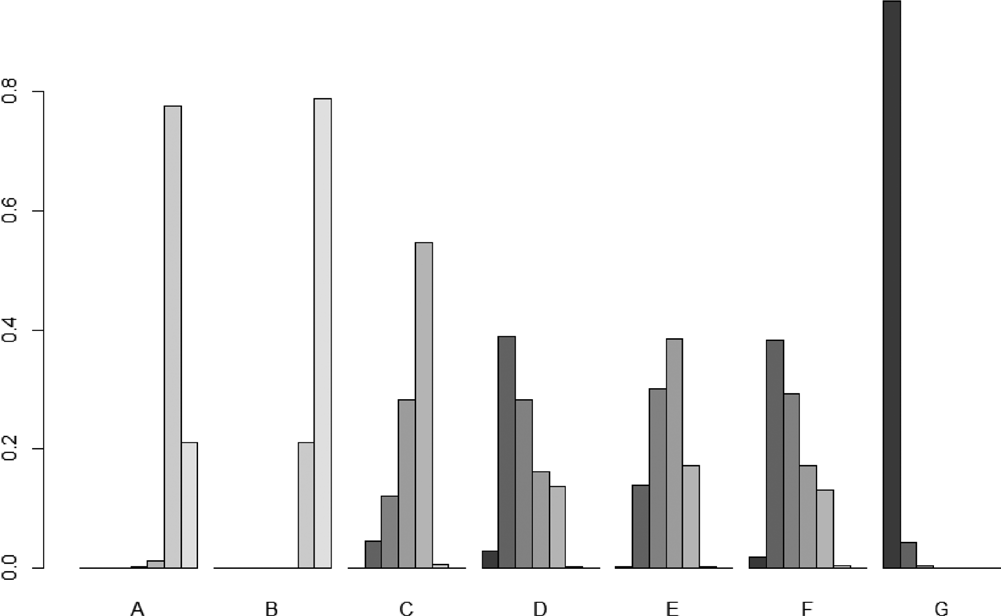
Efficient SUCRA histogram. Notes: The shade of color indicates the likelihood of the intervention being ranked separately, the darker the color the higher the likelihood of the intervention being the preferred treatment.

The SUCRA chart and SUCRA numerical table were developed for the effective rate of different intervention measures for KOA treatment. The effective SUCRA values of the 7 acupuncture treatment measures were ranked from highest to lowest as follows: silver needle, floating needle, needle knife, fire needle, warm needle, conventional acupuncture, and electroacupuncture. Silver needle had the best intervention, as shown in Figure 5 and Table 5.

**Table 5:** SUCRA values for the efficiency of each intervention.

A	B	C	D	E	F	G
0.133860	0.035302	0.442006	0.667304	0.567981	0.662443	0.991102

A: electroacupuncture; B: acupuncture; C: warm needling; D: floating needle; E: fire needle; F: needle knife; and G: silver needle.

#### 3.4.5. Publication bias.

**Figure 6.**
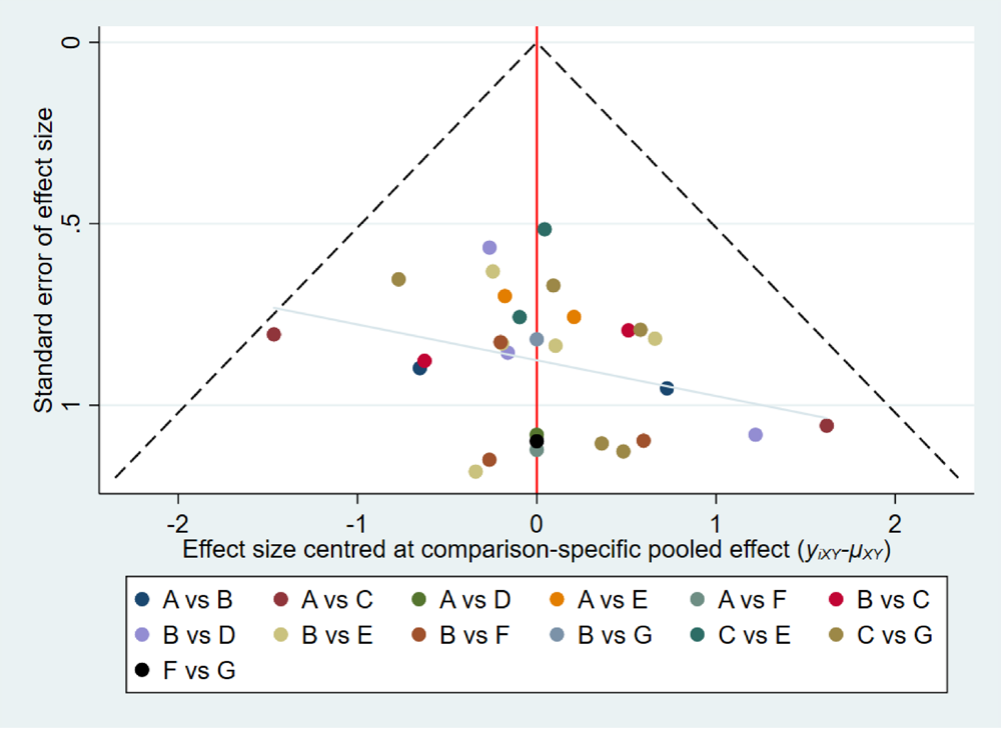
Comparative-corrected funnel plot of Efficient.

The overall efficiency “comparison-corrected” funnel plot exhibited satisfactory symmetry, indicating a low likelihood of publication bias among the included studies, as depicted in Figure 6.

### 3.5. VAS score

#### 3.5.1. Evidence network.

**Figure 7.**
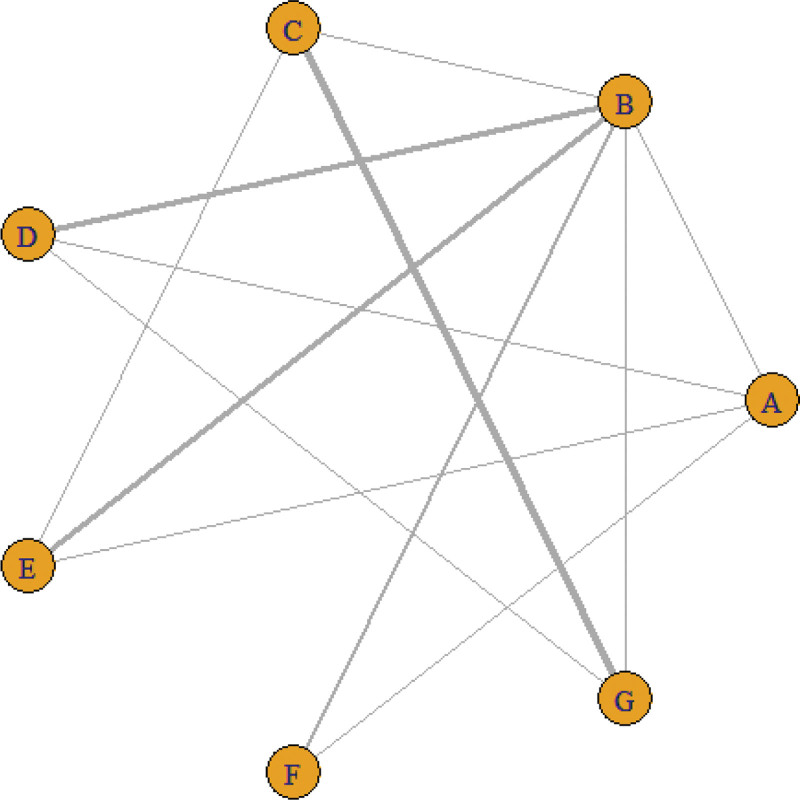
Visual analog scale (VAS) scoring network relationship diagram. Notes: The thicker and darker the line, the greater the correlation between the 2 studies.

Twenty-five studies have reported treatment VAS scores involving 7 acupuncture methods. The network relationship between the interventions is shown in Figure 7. The results demonstrated that the number of studies on the comparison between the silver needle and warm needle treatment was the highest (5 RCTs), and the number of studies with the largest sample size was silver needle versus warm needle (432 cases), See Table 6 for details.

**Table 6:** Visual analog scale (VAS) score network evidence table.

Interventions		Sample size		Average age/yr		Treatment/wk
T	C	T	C	T	C	
A	B	71	67	59.19 ± 5.75	59.20 ± 7.66	4
A	C	93	94	37.35 ± 10.83	38.86 ± 12.12	8
D	B	129	128	58.80 ± 4.48	59.13 ± 4.56	2
D	A	50	50	53.21 ± 7.65	52.21 ± 6.65	3
E	B	159	160	57.12 ± 2.64	57.72 ± 1.33	2
A	E	115	117	64.8 ± 6	64 ± 6	4
E	C	94	94	51.16 ± 2.06	51.14 ± 2.04	4
G	F	35	35	70.21 ± 0.87	70.12 ± 0.74	3
C	B	32	32	60.5 ± 5.4	61.1 ± 5.3	6
G	C	216	216	52.10 ± 5.04	52.32 ± 5.15	3
G	B	40	40	62 ± 5	65 ± 5	3
F	B	101	100	57.20 ± 8.32	58.87 ± 9.63	3
F	A	25	25	56.4 ± 6.1		2

A: electroacupuncture; B: acupuncture; C: warm needling; D: floating needle; E: fire needle; F: needle knife; G: silver needle.

#### 3.5.2. Direct comparison among meta-analysis results and consistency tests.

**Figure 8.**
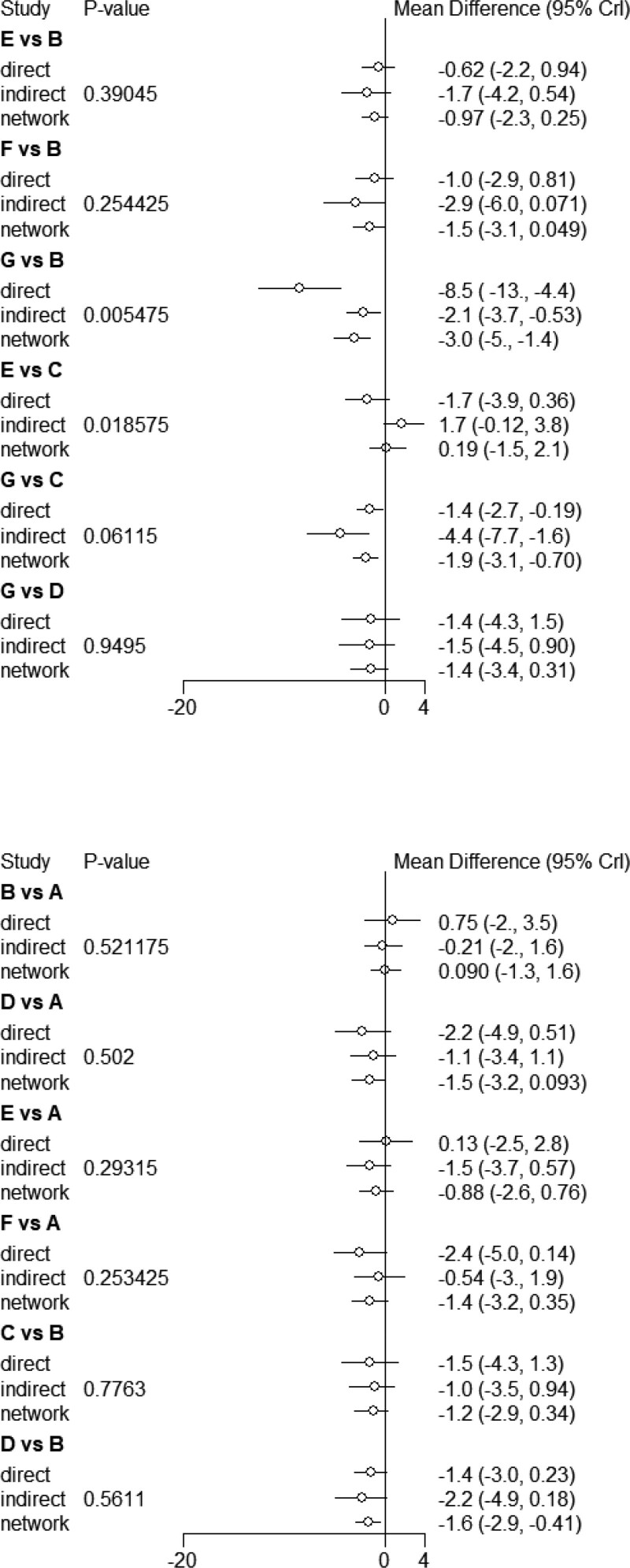
Direct comparison of visual analog scale (VAS) scores and inconsistency tests.

A meta-analysis was performed on the literature that was compared in pairs with the effective rate as the main index. The results were as follows: conventional acupuncture was superior to fire needle (OR = −0.62, 95% CI [2.2, 0.94]), conventional acupuncture was superior to acupotomy (OR = −1.0, 95% CI [2.9, 0.81]), conventional acupuncture was superior to silver needle (OR = −8.5, 95% CI [−13, −4.4]), warm needle was superior to electroacupuncture (OR = −1.7, 95% CI [−3.9,0.36]). Warm needle was better than silver needle (OR = −1.4, 95% CI [−2.7, −0.19]), float needle was better than silver needle (OR = −1.4, 95% CI [−4.3, 1.5]). The results demonstrated a statistically significant difference between the direct and indirect comparison of silver needle and conventional acupuncture and fire needle and warm needle (*P* < .05), as well as local inconsistency. The results are shown in Figure 8 and Table 7.

**Table 7:** Visual analog scale (VAS) scoredirect comparison between different acupuncture methods.

Treatment group	Control group	OR	95% Confidence interval
Conventional acupuncture	Fire needle	−0.62	95% CI (2.2, 0.94)
Conventional acupuncture	Acupotomy	−1.0	95% CI (2.9, 0.81)
Conventional acupuncture	Silver needle	−8.5	95% CI (−13, −4.4)
Warm needle	Electroacupuncture	−1.7	95% CI (−3.9, 0.36)
Warm needle	Silver needle	−1.4	95% CI (−2.7, −0.19)
Float needle	Silver needle	−1.4	95% CI (−4.3,1.5)

#### 3.5.3. Reticulation meta-analysis.

A total of 25 articles involved VAS scores. The results of the network meta-analysis of VAS under the consistency model were as follows: electroacupuncture was better than silver needle (OR = 2.94, 95%CI [0.94,5.26]), conventional acupuncture was better than float needle (OR = 1.6, 95% CI [0.40,2.94]), conventional acupuncture was better than silver needle (OR = 3.03, 95%CI [1.47, 4.97]), warm needle was better than silver needle (OR = 1.87, 95% CI [0.72,3.14]). Fire needle was superior to silver needle (OR = 2.07, 95% CI [0.29, 4.18]), whereas the remaining comparisons were not statistically significant (see Table 8 for details).

**Table 8:** Results of the reticulated meta-analysis of Visual analog scale (VAS) scores.

A	B	C	D	E	F	G
0.149850	0.099508	0.510150	0.676391	0.447700	0.640350	0.976050

The shade of color indicates the likelihood of the intervention being ranked separately, the darker the color the higher the likelihood of the intervention being the preferred treatment.

A: electroacupuncture; B: acupuncture; C: warm needling; D: floating needle; E: fire needle; F: needle knife; and G: silver needle.

#### 3.5.4. SUCRA probability ranking.

**Figure 9.**
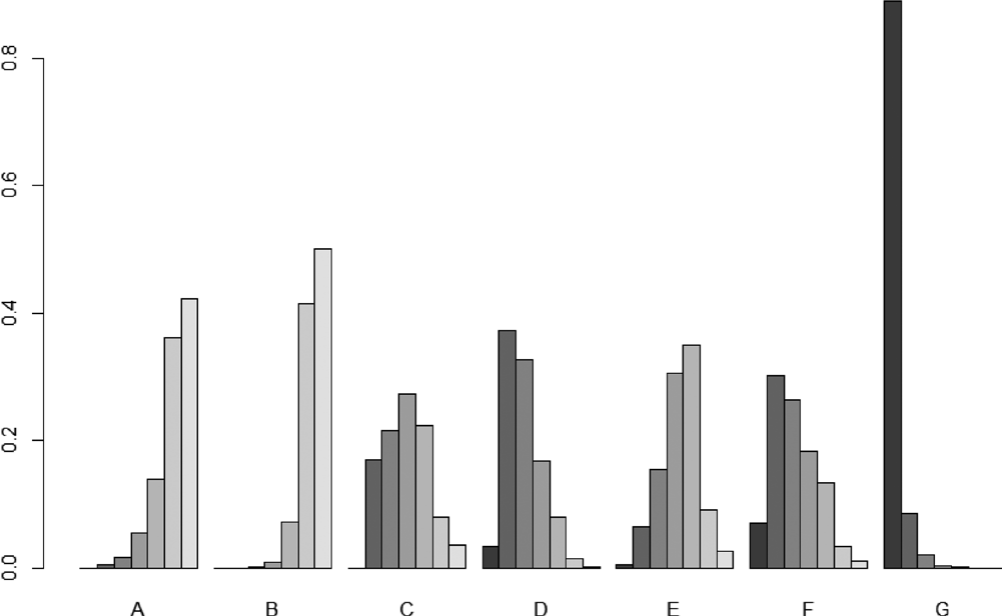
Visual analog scale (VAS) score SUCRA histogram.

The SUCRA value histogram and SUCRA numerical table were developed for the VAS scores of various interventions for KOA treatment. The VAS score of silver needle intervention in all treatment measures was higher than that of other acupuncture treatment schemes. The order of SUCRA values from highest to lowest is as follows: silver needle, conventional acupuncture, needle knife, floating needle, warm needle, fire needle, and electroacupuncture. Silver needle had the best intervention, and the results are shown in Figure 9 and Table 9.

**Table 9:** SUCRA values for Visual analog scale (VAS) scores for each intervention.

A	B	C	D	E	F	G
0.149850	0.099508	0.510150	0.676391	0.447700	0.640350	0.976050

The shade of color indicates the likelihood of the intervention being ranked separately, the darker the color the higher the likelihood of the intervention being the preferred treatment.

A: electroacupuncture; B: acupuncture; C: warm needling; D: floating needle; E: fire needle; F: needle knife; and G: silver needle.

#### 3.5.5. Publication bias.

**Figure 10.**
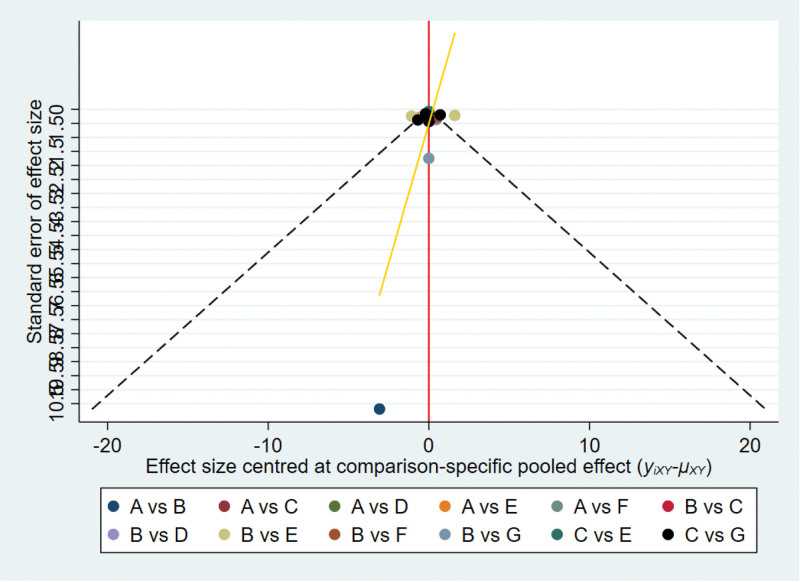
Comparative-corrected funnel plot of visual analog scale (VAS). (A) Electroacupuncture; (B) acupuncture; (C) warm needling; (D) floating needle; (E) fire needle; (F) needle knife; (G) silver needle; ①: efficiency rate; ②: VAS score; T: treatment group; C: control group.

Comparative-corrected funnel plots were generated for every outcome indicator in the studies included, and the ensuing plots exhibited a higher concentration of data points, suggesting reduced data variability. This is visually demonstrated in Figure 10.

## 4. Discussion

KOA is easy to cause chronic disability in the clinical population. It is an age-related degenerative OA disease. The incidence group consisted primarily of middle-aged and elderly people. The main symptoms of KOA are knee stiffness, pain, and movement disorders, which have a detrimental effect on patients’ quality of life.^[46]^ The present treatment commonly used in western medicine has the risk of causing adverse reactions. A large number of acupuncture and moxibustion techniques have been applied to the clinical practice of KOA and have achieved good clinical efficacy; this is also an important reason why acupuncture and moxibustion therapies can be widely promoted because of their low side effects and high compliance of patients.^[47]^ However, the direct comparative evidence of different acupuncture methods was lacking, so the guidelines did not recommend the optimal acupuncture protocol. To provide clinical guidance for the acupuncture treatment of KOA, this study used network meta-analysis for the first time to compare and sort the relevant efficacy indicators of acupuncture treatment of KOA.

Through network meta-analysis, various common clinical acupuncture treatment schemes for KOA were comprehensively and quantitatively compared. This study encompassed a total of 2488 patients and investigated 7 treatment schemes, namely conventional acupuncture, electroacupuncture, warm needle, fire needle, floating needle, needle knife, and silver needle. The findings offer valuable insights for clinical decision-making in KOA treatment. The results notably highlight the efficacy of silver needle therapy, which demonstrated the ability to reduce the VAS score. Silver needle therapy presents clear advantages for KOA treatment and can be considered as a prioritized clinical intervention. Given the information presented, this study holds relevance for both scientific research and healthcare practitioners involved in the selection of acupuncture and moxibustion therapies for KOA.

The silver needle represents an evolved therapeutic approach derived from traditional acupuncture, integrating the benefits of acupuncture and moxibustion. Clinical investigations have provided evidence that combining acupuncture with heated needle handles can significantly improve local blood circulation, reduce muscle spasms, alleviate pain, and decrease levels of interleukin-6 (IL-6), interleukin-8 (IL-8), and tumor necrosis factor-alpha (TNF-α). The therapeutic efficacy of the silver needle stems from the introduction of heat, generated through moxibustion, into deeper tissues via its thermal conductivity. Various scholars have demonstrated that the silver needle can regulate the release of vasoactive substances by improving local microcirculation in injured muscles, thereby achieving the goal of relieving muscle spasms.^[48]^ Additionally, the densely arranged silver needle maximizes the needle-knife effect, facilitating the loosening of adhesions and rapidly enhancing knee joint function.^[49]^

This study employed network meta-analysis to compare the clinical effectiveness of different acupuncture treatments for KOA. The study incorporated a considerable number of studies with a large sample size, yielding statistically significant outcomes. However, certain limitations should be acknowledged: The included studies exhibited an average quality, and there was heterogeneity in terms of disease duration, age, and treatment duration among the participants, potentially influencing the results. The absence of standardized clinical efficacy criteria across studies introduces some instability to the findings. The indirect nature of the included studies and the lack of direct comparisons between various acupuncture treatment modalities may lead to controversial analysis results. Given the relatively short treatment duration in the included studies, long-term efficacy for KOA remains unexplored. Consequently, further confirmation of these findings necessitates additional high-quality, multicenter, and large-scale RCT.

In conclusion, silver needle therapy exhibits significant superiority in enhancing the total effective rate of KOA treatment, improving knee joint function, and alleviating symptoms. With its substantial clinical value, this therapy holds great potential as the primary option for clinical management of KOA.

## Author contributions

**Conceptualization:** Weiwei Ma.

**Data curation:** Wei Cheng.

**Funding acquisition:** Wei Cheng.

**Resources:** Xin Huang.

**Software:** Xin Huang.

**Writing – original draft:** Weiwei Ma.

**Writing – review & editing:** Weiwei Ma, Chao Yang Zhang.
